# The effect of ‘Zesy002’ kiwifruit (*Actinidia chinensis* var. *chinensis*) on gut health function: a randomised cross-over clinical trial

**DOI:** 10.1017/jns.2019.14

**Published:** 2019-05-03

**Authors:** Sarah L. Eady, Alison J. Wallace, Christine A. Butts, Duncan Hedderley, Lynley Drummond, Juliet Ansell, Richard B. Gearry

**Affiliations:** 1 The New Zealand Institute for Plant & Food Research Limited, Lincoln, New Zealand; 2 The New Zealand Institute for Plant & Food Research Limited, Palmerston North, New Zealand; 3 Drummond Food Science Advisory Limited, Christchurch, New Zealand; 4 Zespri International Ltd, Mount Maunganui, New Zealand; 5 Department of Medicine, University of Otago, Christchurch, New Zealand

**Keywords:** Kiwifruit, Metamucil, Constipation, Gastrointestinal comfort, Complete spontaneous bowel movements, BSS, Bristol Stool Scale, CBM, complete bowel movement, CSBM, complete spontaneous bowel movement, FC, functional constipation, GSRS, Gastrointestinal Symptom Rating Scale, IBS, irritable bowel syndrome, IBS-C, irritable bowel syndrome complicated with constipation, IBS-SSI, Irritable Bowel Syndrome Symptom Severity Index, SBM, spontaneous bowel movement

## Abstract

Functional gastrointestinal disorders including constipation affect up to 14 % of the world's population. Treatment is difficult and challenging resulting in a need for alternative safe and effective therapies. The present study investigated whether daily consumption of three gold-fleshed kiwifruit could alleviate constipation and improve gastrointestinal discomfort in mildly constipated individuals with and without pain. A total of thirty-two participants were enrolled in a 16-week randomised, single-blind, crossover study. Participants received either three ‘Zesy002’ kiwifruit or 14·75 g Metamucil^®^ (5 g dietary fibre/d (a positive control)) for 4 weeks each with a 4-week washout between treatments. A 2-week washout period was included at the beginning and end of the study. Daily bowel habit diaries were kept throughout the study. The primary outcome measure was differences in the number of complete spontaneous bowel movements (CSBM). Secondary outcome measures were bowel movement frequency and stool form as well as digestive symptoms and comfort. The number of CSBM per week was significantly greater during daily consumption of three kiwifruit compared with the baseline (6·3 *v.* 3·3; *P* < 0·05) and the Metamucil^®^ treatment (6·3 *v.* 4·5; *P* < 0·05). Stool consistency was also improved, with kiwifruit producing softer stools and less straining (*P* < 0·05). Gastrointestinal discomfort was also improved compared with baseline for abdominal pain, constipation and indigestion (*P* < 0·05) during the kiwifruit intervention and constipation during the Metamucil^®^ intervention (*P* < 0·05). This randomised controlled trial demonstrates that daily consumption of three gold-fleshed kiwifruit is associated with a significant increase of two CSBM per week and reduction in gastrointestinal discomfort in mildly constipated adults.

Bowel disorders (functional diarrhoea, functional constipation (FC), irritable bowel syndrome (IBS) with predominant diarrhoea, IBS with predominant constipation (IBS-C) and IBS with mixed bowel habits) are common conditions in the general population^(^^1^^)^. These disorders do not always occur as single entities and are thought to occur as part of a continuum with overlap of symptoms which include episodes of bloating, abdominal pain/cramp and borborygmi^(^^2^^)^. As such, the diagnosis of these conditions is complex but is based on the diagnostic criteria outlined in the Rome IV criteria which define these disorders as ‘disorders of the gut–brain interaction classified by gastrointestinal symptoms relating to any combination of motility disturbance, visceral hypersensitivity, altered mucosal and immune function, gut microbiota and/or central nervous system processing’^(^^2^^)^. As one of these bowel disorders, constipation is highly prevalent with up to 14 % of the general population reporting with the condition^(^^3^^)^. It is characterised by having at least two of six symptoms (straining, lumpy/hard stools, incomplete evacuation, sensation of anorectal obstruction, need for manual manoeuvres and fewer than three bowel movements per week that are infrequent and hard to pass), whilst IBS-C is also complicated by pain, which can be severe and frequent^(^^2^^,^^4^^)^. Sufferers can often be seriously impaired by the effects of these conditions, resulting in a significant impact on their quality of life^(^^5^^)^. Many factors contribute to the development of constipation, including age, diet, lifestyle, use of certain medications, psychological and neurological conditions. It is also more common in females than males^(^^1^^)^.

Only one-third of sufferers of constipation seek medical care^(^^6^^,^^7^^)^. The multiple symptoms that are part of the condition make it difficult to manage for sufferers, with current medical therapies such as laxatives, stool softeners and bulking agents having unpleasant side effects that may leave patients dissatisfied. Therapies including fibre supplements such as psyllium are frequently used treatments for constipation and have been shown to be effective^(^^8^^)^. Clinical trials show that compared with a placebo or some laxative therapies, consumption of psyllium leads to an increase in stool frequency and softer stool consistency^(^^8^^–^^10^^)^. It may, however, also lead to an increase in gas or bloating, and the texture/taste of the products may not always appeal to consumers^(^^11^^)^. Other remedies, such as over-the-counter laxatives, different fibre supplements, and lifestyle changes such as increasing water intake and exercise, may not have the clinical evidence supporting their effectiveness^(^^8^^)^. Consequently, effective natural, food-based, convenient alternatives are needed and desired.

A study of forty participants by Attaluri *et al*.^(^^12^^)^ demonstrated that dried plums are one such convenient food product that is an effective treatment for constipation. In that study, consumption of 50 g/d dried plums resulted in a significant increase in stool frequency and softer consistency (*P* < 0·05) as well as an increase in subjective measures rating gastrointestinal comfort, compared with 11 g/d psyllium or pre-baseline measurements. In other studies, green-fleshed kiwifruit (*Actinidia chinensis* var. *deliciosa* ‘Hayward’) has also been identified as a whole food alternative treatment^(^^13^^)^. Anecdotally, kiwifruit are reported as being effective in assisting with the relief of constipation, with some clinical studies supporting this by reporting a significant reduction in abdominal discomfort in constipated individuals following the consumption of green kiwifruit^(^^13^^)^. The effect is largely attributed to the soluble fibre content of the fruit, which has a high water-holding capacity (ability to incorporate water into the fibre matrix), and high viscosity that helps with faecal bulking and softening^(^^14^^)^. Further digestive health efficacy may also be related to the activity of actinidin, a proteolytic enzyme that may produce laxative effects, and a high polyphenol content that may also confer further health benefits^(^^15^^)^. Previous clinical trials investigating the performance of ‘Hayward’ kiwifruit in healthy individuals as well as in older persons, constipated and IBS-C populations showed significant improvements relating to digestive health, including increased faecal frequency and reductions in bowel transit times as well as positive changes in overall wellbeing^(^^16^^–^^18^^)^. Furthermore, no unpleasant side effects have been documented. Whilst the clinical evidence for the efficacy of green-fleshed kiwifruit regarding gastrointestinal comfort and laxation is growing, there are no data concerning the gastrointestinal effects of gold-fleshed kiwifruit. *A. chinensis* var. *chinensis* ‘Zesy002’ is a cultivar of gold-fleshed kiwifruit that has been more recently commercialised as Zespri^®^ SunGold Kiwifruit. It has a slightly different nutritional profile from green-fleshed kiwifruit in that it contains half the total fibre of the green cultivar (1·5 g/100 g fruit as opposed to 3 g/100 g fruit) but the same amount of soluble fibre. It also has less activity of the enzyme actinidin (26 *v*. 100 % activity). It is, however, higher in vitamin C (160 *v*. 85 mg/100 g) and contains similar concentrations of other vitamins (such as folate), phytochemicals and minerals, making it a valuable nutrient-dense food^(^^19^^)^.

We designed a randomised, single-blinded, positively controlled, cross-over study to examine if *A. chinensis* var. *chinensis* ‘Zesy002’ is effective in increasing bowel movement frequency as measured by complete spontaneous bowel movements (CSBM) and provide relief from other symptoms of gastrointestinal discomfort. The study compared the effect of kiwifruit with a baseline period of no kiwifruit consumption and with that produced by a well-recognised over-the-counter laxative therapy, Metamucil^®^, in the treatment of adults with mild constipation. Our aims were to determine the efficacy of three ‘Zesy002’ kiwifruit as a food-based treatment for the relief of constipation and gastrointestinal discomfort in mildly constipated adults.

## Materials and methods

### Ethics

This study was conducted according to guidelines laid down in the Declaration of Helsinki and all procedures involving human subjects were approved by the New Zealand Human Disability and Ethics Committee (16STH86). All participants gave written informed consent. The trial was registered with the Australia New Zealand Clinical Trials Registry (ACTRN: 12616000775415p; http://www.anzctr.org.au).

### Participants

A total of thirty-five participants were recruited through newspaper and radio advertisements, local district health boards and tertiary institution newsletters, existing participant databases and community adverts. Participants were aged between 18 and 65 years, and had a BMI between 18 and 35 kg/m^2^. Fasting blood glucose concentration was required to be below 6·0 mmol/l. Exclusion criteria included the presence of alarm features associated with bowel habit such as weight loss, rectal bleeding, recent changes in bowel habit (>3 months), abdominal pain, occult blood in stools, anaemia, anal fissures and bleeding haemorrhoids. Participants were also excluded if they had severe IBS-C on the Irritable Bowel Syndrome Symptom Severity Index (IBS-SSI) questionnaire. Additional exclusion criteria included a known allergy or sensitivity to kiwifruit and/or psyllium, inflammatory bowel disease, diarrhoea, previous gastrointestinal surgery (not including appendectomy or cholecystectomy), severe CVD, chronic renal failure, pregnancy or planning to become pregnant in the 3 months following the trial period, and neurological conditions such as multiple sclerosis, spinal cord injury and stroke. Participants diagnosed with conditions requiring the use of selective serotonin reuptake inhibitors, tricyclic antidepressants, opiates or anti-inflammatories were eligible for the study if the condition was stable and the medication had been in use continually at a stable dose for greater than 3 months.

### Study design

**Fig. 1.**
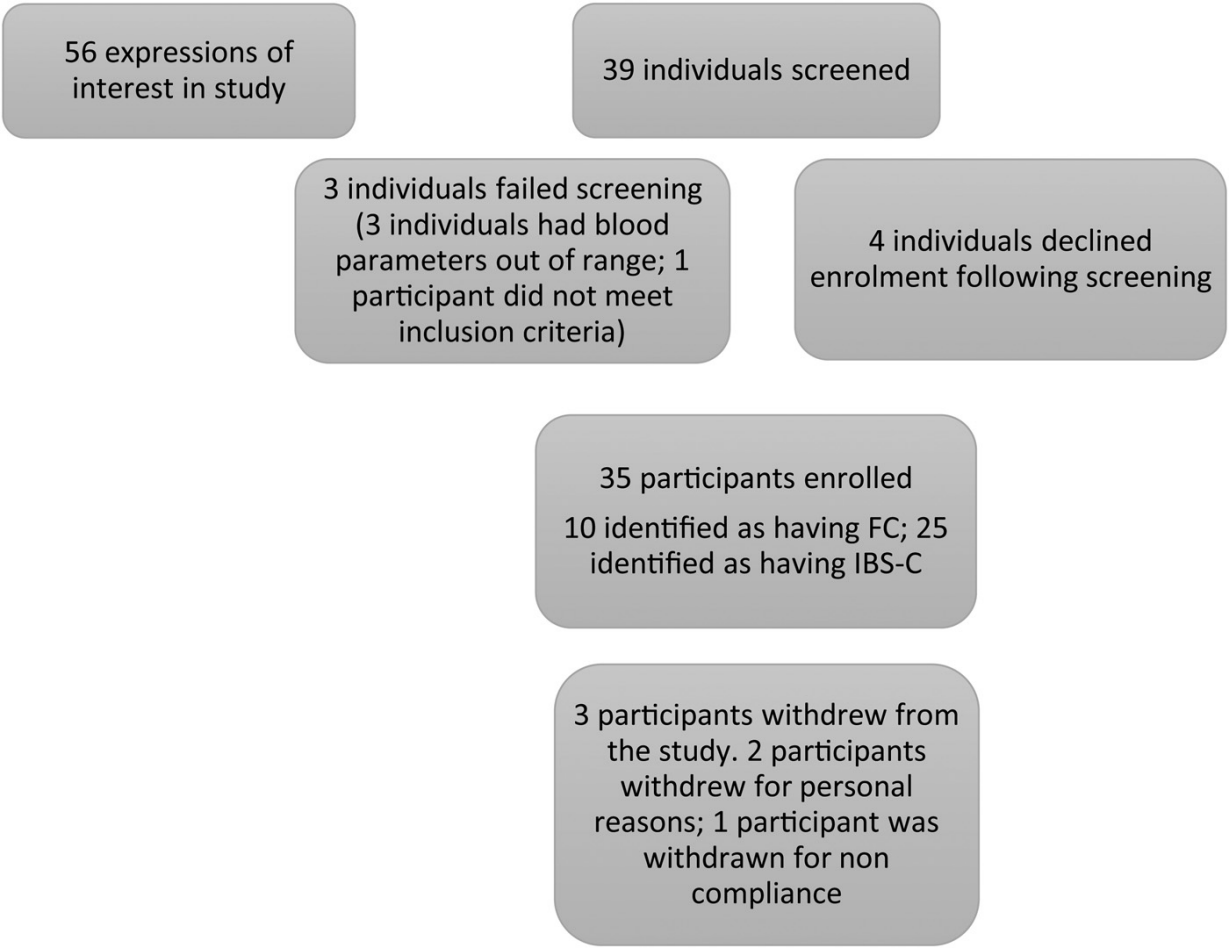
Diagram showing study enrolment and reasons for screen exclusion. FC, functional constipation; IBS-C, irritable bowel syndrome complicated with constipation.

Subjects were enrolled into a 16-week single-blinded randomised cross-over study (Fig. 1). All subjects underwent a comprehensive clinical evaluation before commencing the study including collection of anthropometric data and a Chem 20 blood biochemistry panel. Participants were classified as having either FC or IBS-C as defined by the Rome III criteria (Rome Foundation, USA). IBS severity was assessed using the IBS severity score^(^^18^^,^^20^^)^. Individuals were asked to continue with their habitual diet and lifestyle but were requested to exclude kiwifruit, high-fibre supplements and laxatives (except for a prescribed rescue laxative, Bisacodyl) for at least 2 weeks prior to starting the study and during the course of the trial period. A total of thirty-nine participants were screened for eligibility into the study. An initial lead-in period of 14 d was undertaken before the start of the study during which time the participants maintained a daily bowel habit diary to record the number and nature of the bowel motions and faecal characteristics. Participants were then randomised to receive either three ‘Zesy002’ (Zespri^®^ SunGold Kiwifruit) per d (Zespri International Ltd) or 2·5 teaspoons of Metamucil^®^ (Proctor & Gamble Australia Pty Ltd) providing 5 g of fibre per d which is equivalent to the amount of fibre contained in three ‘Zesy002’ kiwifruit (1·5 g dietary fibre/100 g fruit). Study personnel involved with the participants were blinded to the order of interventions, which were allocated by a biostatistician using computer-generated random numbers. The kiwifruit were delivered to the participants on a weekly basis.

The Metamucil^®^ was supplied in one 283 g container and participants were advised to discard any remaining product following the completion of the intervention to avoid any further consumption. Participants were able to consume the kiwifruit however they liked but they were asked not to consume the skin of the fruit or to blend the fruit into smoothies. They were also able to consume the Metamucil^®^ however they liked but they were asked not to heat it directly, and to consume the entire 2·5 teaspoons at one time. Accurate medicine spoons were supplied to each participant to ensure that equal amounts of Metamucil^®^ were taken. Participants were advised to take two and a half level medicinal teaspoonful full per d, which equated to 14·5 g Metamucil^®^ powder per d. They were also advised to drink a glass of water with the Metamucil^®^. The intervention period was for 4 weeks, after which time participants entered into a washout phase of 4 weeks before being crossed over to receive the alternate intervention for a further 4-week period. Participants were asked to maintain the daily bowel habit diary for the entire study period. A rescue laxative (5 g Bisacodyl suppositories) was available upon request for participants experiencing extreme episodes of constipation during the study; however, if participants did resort to using other laxatives during the study they were asked to record it in their daily diaries. A final washout phase of 14 d occurred following the second intervention.

### Measurements

Participants were required to keep a daily bowel habit diary which included questions about the number and nature of their bowel movements, including the spontaneity and completeness of the bowel movements, rate of laxative use, manual manoeuvres, straining and assessing stool form with the Bristol Stool Scale (BSS). In addition, participants completed the Gastrointestinal Symptom Rating Scale (GSRS; AstraZeneca Ltd) on a weekly basis. The GSRS is a validated outcome measure which rates constipation-related symptoms on a seven-point Likert scale (from 1: no discomfort at all to 7: very severe discomfort)^(^^21^^)^. At the beginning and end of each treatment phase, participants were asked to complete a validated Rome III criteria for constipation questionnaire as used in screening. In addition, participants were also asked to provide a faecal sample and venous blood sample. Faecal samples were kept frozen at −80°C for analysis at a later date for DNA sequencing of faecal bacterial populations. Venous blood samples were analysed for vitamin C content using HPLC, with electrochemical detection as described previously as a measure of compliance with kiwifruit consumption^(^^22^^)^. Finally, a 3-d diet record was collected at the beginning and end of each treatment phase to evaluate dietary stability across the 16-week study. Mean daily macronutrient intake over the 3 d period was calculated using the dietary programme Foodworks version 8.0 (Xyris Software). The average numbers of serves of fruits, vegetables and meat per d were also calculated.

### Outcomes

The primary outcome for this study was a difference in bowel movement frequency as assessed by an increase in the number of CSBM during the intervention periods. A CSBM is defined as a stool/bowel movement that is not caused by taking rescue medications (laxatives/enema) or physical assistance/manual manoeuvres, which leaves a feeling of complete evacuation.

The secondary outcomes for this study were a difference in bowel movement frequency assessed by spontaneous bowel movements (SBM) and complete bowel movements (CBM). SBM is defined as a stool/bowel movement that is not caused by taking rescue medications (laxative/enema) or physical assistance/manual manoeuvres and CBM is defined as a stool/bowel movement which leaves a feeling of complete evacuation and may have been instigated by laxatives or physical assistance. In addition, stool consistency according to the BSS (stool types 1 and 2 indicate hard stools; types 3, 4 and 5 are considered normal consistency, and types 6 and 7 are classified as diarrhoea) was also assessed. Symptoms of gastrointestinal discomfort were measured as a secondary outcome using the GSRS and classified into five domains of diarrhoea, indigestion, constipation, abdominal pain and reflux. The Rome III questionnaire was used to monitor changes in the classification of participants as FC or IBS-C across the 16-week study period.

### Sample size calculation

Using data from an unpublished study (RB Gearry, SL Eady, AJ Wallace, H Dinnan, B Kuhn-Sherlock and CA Butts, unpublished results) carried out in Europe in 2014, it was determined that to detect an increase in CSBM of 1·0 per week in an intervention group receiving Zespri^®^SunGold kiwifruit compared with a control group receiving Metamucil^®^, with 80 % power and 5 % significance, a total of thirty-seven subjects would be required to complete the trial. To allow for a potential 25 % dropout rate, it was aimed to recruit fifty participants to the trial. Because of the limited season of Zespri^®^ SunGold kiwifruit, recruitment was stopped following the enrolment of thirty-five participants because a study of longer duration would have required kiwifruit to be available after the end of the commercial season, which would have affected fruit composition and quality and, subsequently, may have affected the study results. However, the drop-out rate was much lower than anticipated: thirty-two participants completed the study, giving a power of 75 %.

### Statistical analysis

The study was analysed on a per-protocol basis using data from participants who completed the study. Results from the 2-week lead-in period were compared with the results from the ends of the two intervention periods using ANOVA, with person as a random effect and ‘intervention’ (lead-in, kiwifruit or Metamucil^®^) as a fixed effect. The means of the kiwifruit and psyllium (Metamucil^®^) intervention data were compared with the lead-in period and with each other using least significant differences (at *P* = 0·017 to allow for multiple comparisons). Residuals were inspected to ensure that the assumptions of ANOVA were met; where they were not, appropriate transformations were used. All means and standard deviations quoted are based on the raw data.

The GSRS questionnaire was completed each week; the average of the week 1 and week 2 results was used as the lead-in value, and the results from weeks 6 and 14 as the ‘end of intervention period’ values. Similarly, the bowel habit diary was completed daily; the results for the first 2 weeks were averaged to give a per-week value for the lead-in period, and the results from the last 7 d on each intervention were used to calculate per-week ‘end of intervention period’ values. The counts (number of bowel movements, number of CBM, number of SBM, and number of strained bowel movements) were log-transformed for ANOVA, to stabilise the variance; zeros were replaced with 0·5.

The IBS-SSI questionnaire was completed once, at the start of the trial. The results from the four 100-mm line scales from the IBS-C and FC groups were compared using non-parametric Mann–Whitney tests, since the constipated group had skewed data with many zero or low scores. Analysis was done in GenStat (version 16, 2013; VSNi Ltd).

## Results

### Subject characteristics

A total of thirty-two participants completed the study (91 % of those enrolled). Three participants withdrew: two withdrawals were for personal reasons and one participant was withdrawn for non-compliance including not consuming any of the study products and not completing any of the required daily questionnaires resulting in no data for this participant. The demographics of the participants are shown in Table 1. There were no significant differences in the baseline blood biochemistry as analysed by the Chem-20 panel. Self-reported compliance with consumption of the study products was 90 % and the products were well tolerated, with no reports of adverse events relating to the product.

**Table 1.** Baseline characteristics and demographics of study participants (Numbers of participants; medians and ranges)

	Median	Range
Number of participants	32	
Male	0	
Female	32	
Ethnicity (*n*)		
New Zealand European	24	
Maori	2	
Indian	1	
Japanese	1	
Russian	3	
Other	1	
Age (years)	51	21–65
Weight (kg)	68	42–92
BMI (kg/m^2^)	25	20–33

The IBS-SSI questionnaire (Table 2) was included for all participants at screening to maintain equitable treatment of all participants. As expected, those with IBS-C reported experiencing abdominal pain and tended to have greater abdominal distention than those with FC that are not symptoms generally associated with the FC condition^(^^4^^)^.

**Table 2.** Irritable Bowel Syndrome Symptom Severity Index scores for study participants (Mean values and standard deviations)

	Severity of abdominal pain		Severity of abdominal distension		Satisfaction with bowel habits		Effect of IBS-C on quality of life		Sum	
	Mean	sd	Mean	sd	Mean	sd	Mean	sd	Mean	sd
Constipated (*n* 9)	1·6	3·7	10·7	17·6	32·4	21·6	14·2	21·9	58·8	47·3
IBS-C (*n* 23)	22·1	20·3	25·9	24·4	66·9	16·4	43·8	23·7	158·7	59·3
*P*	0·005		0·082		<0·001		0·004		<0·001	

IBS-C, irritable bowel syndrome complicated by constipation.

### Primary outcome: frequency of bowel movements

**Fig. 2.**
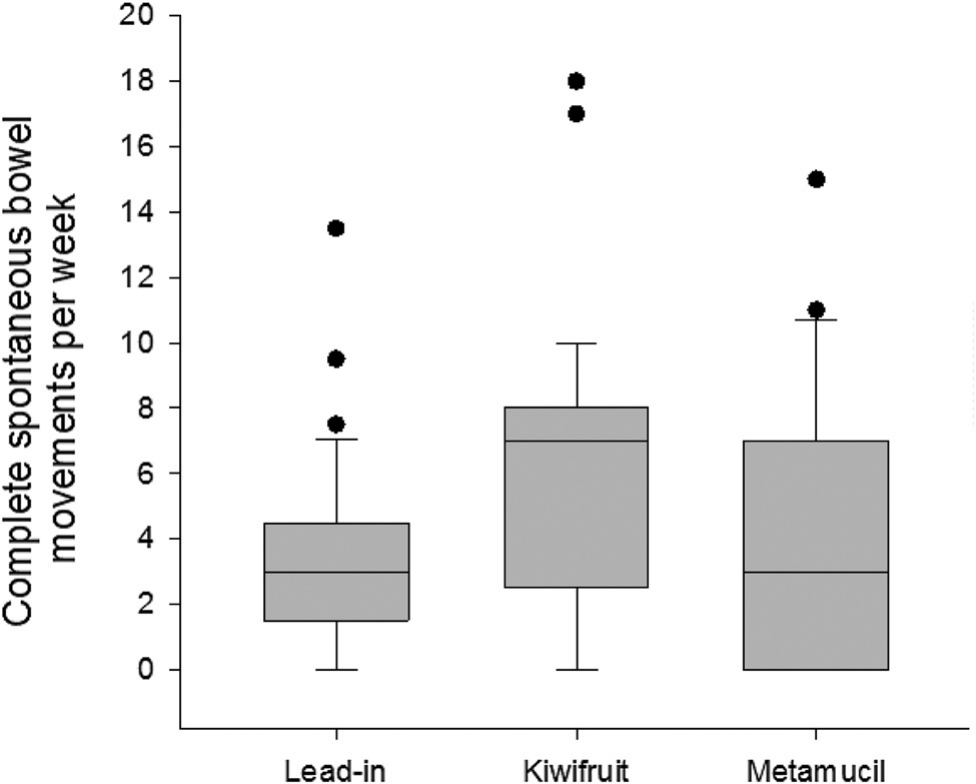
Complete spontaneous bowel movements per week at lead-in period and following intervention. Horizontal lines are medians. The boxes represent interquartile ranges. The whiskers represent ranges. Dots are outliers.

The mean number of CSBM was significantly increased by an average of three CSBM per week between baseline and kiwifruit (3·3 (sd 2·9) *v.* 6·3 (sd 4·5); ANOVA *P* = 0·004). The mean for kiwifruit was also significantly higher than the mean for Metamucil^®^, resulting in an average increase of 1·8 CSBM per week (6·3 (sd 4·5) *v.* 4·5 (sd 4·6)). The difference between baseline and Metamucil^®^ was not significant (3·3 (sd 2·9) *v.* 4·5 (sd 4·6)). The results are shown in Fig. 2.

### Secondary outcomes

#### Stool frequency

There was a significant average increase of 1·9 in the total number of bowel movements (7·2 (sd 3·5) *v.* 9·1 (sd 4·9); *P* < 0·05) and an average increase of 2·7 CBM between baseline and the kiwifruit intervention (4·2 (sd 3·1) *v.* 6·9 (sd 4·3); *P* < 0·05) (Table 3). Compared with baseline, there was a non-significant increase in SBM in participants consuming kiwifruit. There were also non-significant increases in all types of bowel movement frequency from baseline in those consuming Metamucil^®^. Whilst both treatments increased stool frequency from the lead-in period, there were no significant differences observed in the increase in both total number of bowel movements or CBM between the kiwifruit intervention and the Metamucil^®^ intervention.

**Table 3.** Secondary changes to bowel movement frequency (Mean values and standard deviations)

	Lead-in period		Zespri^®^ SunGold kiwifruit		Metamucil^®^		
Parameter	Mean	sd	Mean	sd	Mean	sd	*P*
No. of bowel movements/week	7·2	3·5	9·1*	4·9	8·1	3·8	0·02
No. of CBM/week	4·2	3·1	6·9*	4·3	5·5	4·2	0·002
No. of SBM/week	6·7	3·6	8·6	5·0	7·2	4·5	0·226
Number of strained bowel movements/week	3·7	2·6	1·7*	2·4	2·5*	3·4	<0·001
Average Bristol Stool Scale score	3·24	1·13	4·15*†	1·26	3·52	1·27	<0·001
Proportion of bowel movements using laxatives (%)	2·8	10·9	5·2	20·9	3·4	17·7	‡

CBM, complete bowel movements; SBM, spontaneous bowel movements.

* Mean value was significantly different from that for the lead-in period, based on least significant difference.

† Mean value was significantly different from that for Metamucil^®^, based on least significant difference (*P* = 0·025).

‡ Not analysed. Of the participants, 92 % did not use.

#### Stool consistency and straining

Compared with baseline, there was a significant reduction of an average of 2 BM for the kiwifruit intervention and an average of 1·2 BM for Metamucil^®^ in the number of bowel movements associated with straining (*P* < 0·001). The reduction was not significant between when comparing the two interventions with each other. Following the intervention phase, there was a significant increase in the BSS score indicating softer stool consistency with kiwifruit than at baseline (4·2 *v.* 3·2; ANOVA *P* < 0·001; Table 3). The average BSS score was also significantly higher with kiwifruit than with Metamucil^®^ (4·2 *v.* 3·5).

### Laxative use

Of participants, 92 % did not use laxatives at any point during the study. Of the 8 % (*n* 3) of participants who did require rescue laxatives, 5·2 % were during the kiwifruit intervention, whilst 3·4 % took laxatives during the Metamucil^®^ intervention.

### Overall gastrointestinal symptoms

Participants rated their degree of gastrointestinal discomfort using the GSRS. Fifteen questions relating to gastrointestinal discomfort were rated on a 1–7 scale (no discomfort to very severe discomfort). Following Svedlund *et al*.^(^^21^^)^, the GSRS data were grouped into five subscales or domains (Table 4).

**Table 4.** Gastrointestinal Symptom Rating Scale questionnaire scores for combined functional constipation and irritable bowel syndrome with constipation participant groups (Mean values and standard deviations)

	Lead-in period		Zespri^®^ SunGold kiwifruit			Metamucil^®^		
	Mean	sd	Mean	sd	*P*	Mean	sd	*P*
Diarrhoea	1·53	0·71	1·89	1·51	>0·05	1·44	0·68	>0·05
Indigestion	2·54	0·95	1·87*†	0·80	0·002	2·33	1·02	>0·05
Constipation	3·11	1·41	1·92*	1·16	<0·001	2·41*	1·43	<0·001
Abdominal pain	1·88	0·70	1·57*	0·61	<0·05	1·84	0·91	>0·05
Reflux	1·41	0·65	1·33	0·68	>0·05	1·35	0·57	>0·05

* Mean value was significantly different from that for the lead-in perood, based on least significant difference (*P* = 0·025).

† Mean value was significantly different from that for Metamucil^®^, based on least significant difference (*P* = 0·025).

There were significant improvements in the three domains of constipation, abdominal pain, and indigestion, compared with baseline, after consuming kiwifruit. In contrast, there was a significant improvement from baseline for only the constipation domain for those consuming Metamucil^®^. When comparing the kiwifruit intervention with the Metamucil^®^ intervention, there were no significant differences between the two interventions except for in the indigestion domain, where the kiwifruit treatment significantly improved symptoms of indigestion in relation to Metamucil^®^ (1·87 *v.* 2·33).

### Rome III status

Individuals were classified as FC or IBS-C using the Rome III criteria at initial screening and over the course of the study at each visit. At screening, there were twenty-three people classified as IBS-C and nine classified as FC. Over the course of the study, the classification of participants into the FC and IBS-C groups was variable (Table 5). McNemar's test showed that the test statistic had a value of 0 (*P* = 1·000) and the changes observed were not significant.

**Table 5.** Classification of functional constipation (FC) and irritable bowel syndrome with constipation (IBS-C) by Rome III criteria (Numbers of participants and percentages)

	Screening		Lead-in		After kiwifruit intervention		After Metamucil^®^ intervention	
	*n*	%	*n*	%	*n*	%	*n*	%
Number meeting Rome III criteria for IBS-C	23	72	16	50	13	41	14	44
Number meeting Rome III criteria for FC	9	28	11	34	12	38	12	38

### Compliance

Plasma vitamin C concentrations were also measured at the beginning and end of each intervention period (as a measure of compliance with the kiwifruit intervention). The kiwifruit intervention resulted in a significantly higher plasma ascorbate than in both the lead-in period (71·8 *v.* 60·1; ANOVA *P* < 0·001) and Metamucil^®^ intervention period (71·8 *v.* 52·7).

### Dietary intake

#### Food intake

Measurement of the participants’ habitual dietary intake without the inclusion of the two study products showed there was a significant decrease in total energy intake between the lead-in period (7942 kJ/d) and the kiwifruit intervention (7025 kJ/d; ANOVA *P* < 0·05). Whilst total energy intake decreased with the Metamucil^®^ intervention in comparison with the lead-in period and kiwifruit intervention, it was not significantly different. Total available carbohydrate decreased significantly during the kiwifruit intervention (175 g/d) compared with baseline (206 g/d; ANOVA *P* < 0·05), as did sugar intake (71 *v.* 87 g/d; ANOVA *P* < 0·05). Furthermore, there was a significant decrease in the quantity of PUFA ingested during the kiwifruit intervention (9 g/d) compared with that in the lead-in period (12 g/d; ANOVA *P* < 0·01). Dietary fibre intake decreased significantly during the Metamucil^®^ intervention and the kiwifruit intervention relative to baseline (ANOVA *P* < 0·01). K and Mg intake both decreased significantly from baseline during the Metamucil^®^ intervention and the kiwifruit intervention compared with baseline (ANOVA *P* < 0·01). Overall there were no significant differences in any of the nutrients measured when comparing the kiwifruit intervention and Metamucil^®^ intervention.

Table 6 shows the average daily intake measured using the 3 d diet records for all participants in both intervention periods.

**Table 6.** Average total daily dietary intake (Mean values and standard deviations)

	Lead-in period		Zespri^®^ SunGold kiwifruit		Metamucil^®^		
Nutrient	Mean	sd	Mean	sd	Mean	sd	*P*
Total energy (kJ)	7942	2181	7025*	1899	7440	2515	0·03
Energy, no dietary fibre (kJ)	7731	2138	6867*	1879	7269	2474	0·04
Protein (g)	83	24	78	23	82	25	0·489
Total fat (g)	76	23	66	25	71	31	0·158
SFA (g)	31	14	28	14	29	15	0·554
PUFA (g)	12	6	9*	4	11	5	0·005*
Available carbohydrates (g)	206	74	175*	63	189	72	0·01
Sugar (g)	87	36	71*	29	81	38	0·021
Dietary fibre (g)	25	10	22*	9	21*	8	0·008*
Vitamin C (mg)	103	78	104	110	82	58	0·401
Na (mg)	2440	956	2297	674	2568	1065	0·348
K (mg)	3232	859	2747*	882	2568	1065	0·348
Mg (mg)	431	167	348*	146	353*	157	0·003*
Fe (mg)	12	4	10	3	11	4	0·069

* Mean value was significantly different from that for the lead-in period, based on least significant difference (*P* = 0·025).

#### Intake of fruits, vegetables and meat

During the kiwifruit intervention, the average intake of fruit per d decreased by 0·4 pieces of fruit per d in comparison with baseline (ANOVA *P* < 0·05; Table 7). There were no significant differences for either meat or vegetable intake. When compared with the Metamucil^®^ intervention, there were no significant differences in fruit, meat or vegetable intake during the kiwifruit intervention. The count of serves of fruit per d excludes the kiwifruit supplied for the trial intervention.

**Table 7.** Daily intake of meat, fruits and vegetables during the study (Mean values and standard deviations)

	Fruit (per d)		Meat (per d)		Vegetables (per d)	
Study period	Mean	sd	Mean	sd	Mean	sd
Lead-in	1·4	0·9	1·2	0·5	2·8	1·6
Zespri^®^ SunGold kiwifruit	1·0*	0·9	1·2	0·7	2·4	1·7
Metamucil^®^	1·2	0·8	1·4	0·6	2·3	1·6

* *P* < 0·05.

## Discussion

In this randomised, single-blind, controlled trial the daily consumption of three gold-fleshed kiwifruit was associated with an increase of greater than two CSBM per week when compared with not consuming kiwifruit and of greater than one CSBM when compared with Metamucil^®^. It also improved symptoms of gastrointestinal discomfort in mildly constipated adults when compared with not consuming kiwifruit or when compared with consuming Metamucil^®^ which is recognised as a treatment for constipated individuals.

Measurement of CSBM as an indication of bowel function in constipated individuals is a robust and validated measure which takes into consideration the heterogeneous nature of constipation^(^^12^^)^. Sufferers can experience a range of symptoms and feelings, including frequent incomplete bowel movements with hard stools requiring an excessive amount of straining, which can be misleading when assessing bowel function accurately. An increase of greater than one CSBM per week is considered a clinically significant marker of improved bowel function in symptomatic individuals^(^^23^^)^. This trial is the first randomised controlled single-blinded study examining the effect of ‘Zesy002’ kiwifruit in mildly constipated adults, and provides preliminary evidence that the consumption of three ‘Zesy002’ kiwifruit daily can be considered as a treatment for constipation alongside recognised treatments such as Metamucil^®^ and its efficacy should be investigated further.

In addition to a significant increase in the average number of CSBM per week for the kiwifruit intervention compared with the lead-in period and Metamucil^®^ intervention, consumption of three ‘Zesy002’ kiwifruit daily also showed a significant increase in the occurrence of CBM. There was also a general increase in SBM per week and overall bowel movement frequency. Metamucil^®^ consumption resulted in a non-significant increase in CSBM, CBM and SBM, showing that this intervention is also effective. Three kiwifruit per d equated to 360 g fruit and provided participants with 5 g of fibre per d. Participants were asked to consume 2·5 medicinal teaspoons of Metamucil^®^, which equated to 14·5 g of powder containing 5 g fibre, to match that of three kiwifruit. The daily dosage suggested by the manufacturer of Metamucil^®^ is 1·5 teaspoons between one and three times daily, which would be between 8·9 and 26·5 g of powder, indicating that the dose used in this study is within the range of the recommended dose range.

Excessive straining due to constipation is a common phenomenon and can severely affect an individual's degree of comfort and quality of life^(^^24^^)^. This study demonstrated that consumption of three ‘Zesy002’ kiwifruit was associated with a significant reduction in straining and, whilst Metamucil^®^ also reduced straining, the effect was not as pronounced. This was accompanied by the change in stool consistency during the kiwifruit intervention compared with baseline and Metamucil^®^, resulting in an increased frequency of softer stools as measured by the BSS. The BSS is widely used in clinical trials to categorise stool consistency determined by the different water contents in the stool, which reflect both rate of intestinal transit and water activity^(^^24^^)^.

The study also showed a significant improvement in constipation, abdominal pain and indigestion associated with the consumption of three ‘Zesy002’ kiwifruit as measured by the GSRS. Gastrointestinal discomfort arising from constipation manifests in several different symptoms, which can have considerable impact on an individual's health^(^^25^^)^. The kiwifruit intervention was associated with a significantly reduced incidence of indigestion compared with baseline and this response was also significantly different from the reduction achieved by Metamucil^®^. Abdominal pain was also significantly reduced from baseline with consumption of the kiwifruit. Furthermore, both the kiwifruit and Metamucil^®^ interventions were associated with a significant reduction in constipation compared with baseline.

There are several proposed mechanisms underlying how kiwifruit exerts its effect on constipation and gastrointestinal discomfort, but these are not yet fully elucidated. These are more fully explained in a recent paper by Bayer *et al*.^(^^13^^)^. These mechanisms are largely attributed to the nutrient profile of kiwifruit and the presence of both soluble and insoluble fibre, which is thought to contribute to the beneficial demonstrated effect on laxation. The insoluble fibre has high water retention, whilst the soluble fibre is highly viscous and has a unique high cell wall water-holding capacity causing enhanced viscosity and swelling. This increases faecal bulk and helps to soften stools to assist in the movement of stools along the colon, relieving symptoms of constipation^(^^13^^,^^26^^)^. Kiwifruit soluble fibre is also highly fermentable by the gut microflora, which has been shown to differ in constipated individuals from that of healthy individuals, with a relative decrease in obligate bacteria such as *Lactobacillus*, *Bifidobacterium* and *Bacteroides* and an increase in potentially pathogenic bacteria such as *Firmicutes* and *Clostridium*^(^^26^^)^. These changes may influence intestinal motility, with slower gastric emptying and colonic transit times observed and changes to the secretory functions occurring through modulation of the metabolic environment of the gut^(^^13^^,^^27^^,^^28^^)^. Kiwifruit also contains the proteolytic enzyme actinidin, which is thought to enhance protein digestion and gastric emptying, and may contribute to a reduction in gastrointestinal discomfort^(^^13^^,^^29^^)^.

The Rome criteria have been adopted as the international standard for diagnosis of functional gastrointestinal disorders and are widely recognised within the field^(^^30^^)^. The questionnaire defines the presence of symptoms relating to functional gastrointestinal disorders in an individual over a 12-week period. This study used the tool to monitor changes in the IBS-C status of trial participants on a 4-weekly basis and whilst some changes were observed, these were not statistically significant and not considered to be clinically relevant. Classification into the IBS-C group is determined by the amount of pain experienced by the individual which is the defining criterion that separates FC from IBS-C^(^^31^^)^. Pain has to be present for on average at least 1 d per week for the preceding 3 months and its severity related to defecation. It may also be associated with changes in stool frequency and stool form^(2)^. Symptom patterns in IBS patients are variable over time, with fluctuations in the symptomology common; thus episodes of certain symptoms such as pain can vary in their length and frequency and be unstable over short periods of time^(^^31^^)^. In 4-week periods of measurement, it is likely that this variation would have affected the responses given by our participants, which may account for the fluctuation in the classification of participants into the IBS-C or FC groups.

Dietary intake patterns were also assessed and showed that there was a significant decrease in the overall total energy intake between the baseline and the kiwifruit intervention, which may be attributed to participants adjusting their dietary intake to accommodate the inclusion of 360 g kiwifruit to their habitual intake. Studies have shown that including low-energy-dense foods such as kiwifruit in the daily diet can help individuals reduce their total daily energy intake since individuals eat a fairly consistent amount of food on a daily basis irrespective of the amount of energy the foods may or may not contain; thus overall energy intake is defined by the energy density of individual foods rather than the total amount of food eaten^(^^32^^)^. Inclusion of Metamucil^®^ into the diet also resulted in a reduction of total energy intake although this was not significant. Research has shown that both fruits and fibre have a satiating effect, and inclusion of these items in the diet can result in overall decreased energy intake^(^^33^^)^. Participants reported consuming less total kJ per d than the recommended daily intake although intakes of protein and total fat were within the recommended range^(^^34^^)^. Participants also had higher than recommended intakes of sugar, Na and saturated fat which is consistent with societal trends and common in diets where high quantities of processed food items are available^(^^35^^)^. It is, however, commonly acknowledged that the accuracy of diet records is questionable in that people do not always pay attention to the food that they are eating, do not remember everything, do not know the ingredients of the foods they are listing and underestimate portion size^(^^36^^)^. In general, however, the small number of changes that were observed do not suggest that participants changed their dietary practices significantly over the 4-month study period. The average daily fruit, vegetable and meat intakes were also measured during the study and as expected, the average intake of other fruits during the kiwifruit intervention fell, presumably to compensate for the study product. There were no other significant differences observed in vegetable and meat intake. The participants in this study were free-living individuals and dietary intake was not controlled. These diet records provide only a snapshot of food habits and these self-reporting tools are challenging in their degree of reliability^(^^36^^)^.

The focus of this study was to observe the impact of kiwifruit on gastrointestinal health irrespective of habitual dietary intake although to reduce confounding, participants were asked to avoid foods with a known laxative effect such as prunes, liquorice and probiotic products which may have affected the study results. It is recognised, however, that including a more thorough analysis of the diet would have given a more comprehensive picture of the dietary influence on bowel health. In order to obtain more rigorous information relating to dietary intake, it would require participants to complete further diet records and preferably incorporate accurate weights and ingredient lists of the foods consumed. Taking into account the length of this study and the high level of participant participation required to complete the daily/weekly questionnaires, it was considered that this would raise the level of participant burden and possibly contribute towards non-compliance to an already demanding study protocol. Further studies in this area could address this question.

The palatability and tolerability of the study products were rated as high by participants and no adverse effects relating to either of the study products were reported. Compliance was reported as high, with 91 % of participants reporting that they took the interventions as requested. Plasma vitamin C measurements were also taken to support the subjective reporting of compliance and this showed a significant increase during the kiwifruit intervention period. It must be noted that there were some anomalies in the amounts of plasma ascorbate across the trial, with large changes from one intervention to the next that were difficult to attribute to the study products. Studies^(^^37^^)^ have shown that plasma vitamin C concentrations are easily influenced by certain confounding factors such as body size, smoking, supplement use, bioavailability, nutrient intake, food processing and disease status, which are difficult to control and may inhibit or enhance absorption, affecting the nutrient's circulating concentration. Additionally, measurement of plasma vitamin C in an individual is affected by their initial vitamin C concentrations. It is also more difficult to demonstrate any further effect from supplementation in subjects who have a high or saturating concentration of vitamin C at baseline (50–70 µmol/l)^(^^38^^)^. In our present study, more than 85 % of participants had mean baseline amounts of plasma vitamin C that were greater than 50 µmol/l. The test does remain, however, a further measure of compliance in addition to self-reported consumption of kiwifruit.

This study had several strengths and limitations. The major limitation was the inability to blind subjects to the whole food interventions that they were receiving and, therefore, bias may have been introduced through the preconceived ideas held by individuals relating to the effect of kiwifruit and/or Metamucil^®^ on constipation. Furthermore, while participants were asked to avoid foods known to affect bowel habits, changes in participants’ diets which may have affected the transit of food through the gastrointestinal tract may not have been accounted for in the study.

The small sample size was also a limitation of the present study and may have increased the margin of error. The original intention of the study was to recruit fifty participants to achieve 80 % power with 5 % significance. However, because of difficulty recruiting within the New Zealand kiwifruit season (June–November) and to maintain uniformity of the fruit avoiding seasonal variations that can affect the fruit's characteristics, only thirty-five participants were recruited within this recruitment window. Of these, thirty-two individuals completed. Retrospectively, a *post hoc* power calculation was performed, based on thirty-two participants and demonstrated a power of 75 % with *P* < 0·05 significance to show a difference of one CSBM per week.

The intention of the present study was to determine the efficacy of consuming three ‘Zesy002’ kiwifruit daily in increasing CSBM, stool form, digestive symptoms and comfort in constipated individuals as compared with baseline and an active control product, Metamucil^®^. Analysis of the data was performed using ‘per-protocol’ analysis; hence data from three participants were excluded. Per-protocol analysis is considered to better reflect the effects of a treatment when taken in the optimal manner but may introduce ‘attrition bias’ reducing the similarity amongst the population characteristics when large numbers of participants are removed^(^^39^^)^. Hence this may be considered a limitation of this study. Due to the very small number of participants who did not complete the trial, however, the effect of this type of analysis and the introduction of bias is thought to be minimal.

Recruitment of only female participants to the study also weakened the strength of the study, as it limits the extrapolation of these results to the population as a whole^(^^40^^)^. The female-only cohort was not deliberate and may have occurred for a variety of reasons. Constipation and IBS occur more commonly in women than in men, which may be related to hormonal factors, stress responses, sex-related differences in autonomic and antinociceptive responses, and also psychological responses^(^^40^^,^^41^^)^. Women also tend to approach health professionals more frequently for help relating to health issues in general^(^^42^^,^^43^^)^.

The use of whole fruit was a strength of this study even though it was a limitation with regard to blinding. Use of the whole fruit ensured that any effects observed were due entirely to the fruit and its natural state rather than to any changes that may have occurred during processing or preparation. Studies have shown that whole-fruit consumption can have markedly different effects on health outcomes from consumption of individual components of the fruit, such as fruit juice^(^^44^^)^. The cross-over design also strengthened this trial, as it allowed for each participant to act as their own control, reducing the effect of intra-personal variation which is observed naturally in any individual's bowel habits.

### Conclusions

This randomised, controlled study demonstrated that consumption of three *A. chinensis* var. *chinensis* ‘Zesy002’ (Zespri^®^ SunGold kiwifruit) daily in mildly constipated individuals resulted in a significant increase in two CSBM per week when compared with baseline and the dietary fibre supplement Metamucil^®^. It also reduced gastrointestinal discomfort by improving symptoms of indigestion, constipation and abdominal pain when compared with baseline and significantly improved indigestion when compared with Metamucil^®^. ‘Zesy002’ were also well tolerated, efficacious and safe.

This is the first study assessing the impact of ‘Zesy002’ on stool frequency and gastrointestinal discomfort, and the results suggest that SunGold kiwifruit has some potential as a possible alternative therapy for constipation. Further studies in this area are required to replicate these findings in other populations and to ascertain a deeper understanding of the mechanisms of action that may be driving these results. These will help to confirm and grow the evidence for ‘Zesy002’ kiwifruit as a whole food which can be incorporated into the daily diet as a treatment for improving symptoms in mildly constipated individuals.

## References

[1] Lacy BE, Mearin F, Chang L, (2016) Bowel disorders. Gastroenterology 150, 1393–1407. 10.1053/j.gastro.2016.02.031 27144627

[2] Schmulson MJ & Drossman DA (2017) What is new in Rome IV? J Neurogastroenterol Motil 23, 151–163. 28274109 10.5056/jnm16214 PMC5383110

[3] Suares NC & Ford AC (2011) Prevalence of and risk factors for chronic idiopathic constipation in the community: a systematic review and meta-analysis. Am J Gastroenterol 106, 1582–1591. 21606976 10.1038/ajg.2011.164

[4] Enck P, Lenert J, Smid M, (2016) Functional constipation and constipation – predominant irritable bowel syndrome in the general population: data from the GECCO study. Gastroenterol Res Pract 2016, 3186016. 26880887 10.1155/2016/3186016 PMC4736007

[5] Mugie SM, Benninga MA & Di Lorenzo C (2011) Epidemiology of constipation in children and adults: a systematic review. Best Pract Res Clin Gastroenterol 25, 3–18. 21382575 10.1016/j.bpg.2010.12.010

[6] Lindberg G, Hamid SS, Malfertheiner P, (2011) World Gastroenterology Organisation global guideline: Constipation – a global perspective. J Clin Gastroenterol 45, 483–487. 21666546 10.1097/MCG.0b013e31820fb914

[7] Schmidt FMQ & De Gouveia Santos VLC (2014) Prevalence of constipation in the general adult population: an integrative review. J Wound Ostomy Continence Nurs 41, 70–76. 24378694 10.1097/01.WON.0000438019.21229.b7

[8] Ashraf W, Park F, Lof J, (1995) Effects of psyllium therapy on stool characteristics, colon transit and anorectal function in chronic idiopathic constipation. Aliment Pharmcol Ther 9, 639–647. 10.1111/j.1365-2036.1995.tb00433.x 8824651

[9] McRorie JW, Daggy BP, Morel JG, (2005) Psyllium is superior to docusate sodium for treatment of chronic constipation. Aliment Pharmacol Ther 12, 491–497. 10.1046/j.1365-2036.1998.00336.x 9663731

[10] Ramkumar D & Rao SS (2005) Efficacy and safety of traditional medical therapies for chronic constipation: systematic review. Am J Gastroenterol 100, 936–971. 15784043 10.1111/j.1572-0241.2005.40925.x

[11] Schiller LR (2001) Review article: the therapy of constipation. Aliment Pharmacol Ther 15, 749–763. 11380313 10.1046/j.1365-2036.2001.00982.x

[12] Attaluri A, Donahoe R, Valestin J, (2011) Randomised clinical trial: dried plums (prunes) vs. psyllium for constipation. Aliment Pharmacol Ther 33, 822–828. 21323688 10.1111/j.1365-2036.2011.04594.x

[13] Bayer SB, Gearry RB & Drummond LN (2017) Putative mechanisms of kiwifruit on maintenance of normal gastrointestinal function. Crit Rev Food Sci Nut 58, 2432–2452. 10.1080/10408398.2017.1327841 28557573

[14] Chan A, Leung G, Tong T, (2007) Increasing dietary fibre intake in terms of kiwifruit improves constipation in Chinese patients. World J Gastroenterol 13, 4771–4775. 17729399 10.3748/wjg.v13.i35.4771 PMC4611199

[15] Udani JK & Bloom DW (2013) Effects of kivia powder on gut health in patients with occasional constipation: a randomised double-blind, placebo-controlled study. Nutr J 12, 78. 23758673 10.1186/1475-2891-12-78 PMC3706267

[16] Rush EC, Patel M, Plank LD, (2002) Kiwifruit promotes laxation in the elderly. Asia Pac J Clin Nutr 11, 164–168. 12074185 10.1046/j.1440-6047.2002.00287.x

[17] Chang CC, Lin YT, Lu YT, (2010) Kiwifruit improves bowel function in patients with irritable bowel syndrome with constipation. Asia Pac J Clin Nutr 19, 451–457. 21147704

[18] Kindleysides S, Kuhn-Sherlock B, Yip W, (2015) Encapsulated green kiwifruit extract: a randomised controlled trial investigating alleviation of constipation in otherwise healthy adults. Asia Pac J Clin Nutr 24, 421–429. 26420182 10.6133/apjcn.2015.24.3.15

[19] Zespri (2016) Zespri Varieties. https://www.zespri.com/varieties/sungold (accessed January 2016).

[20] Francis CY, Morris J & Whorwell KK (1997) The Irritable Bowel Severity Scoring System: a simple method of monitoring IBS and its progress. Aliment Pharmacol Ther 11, 395–402. 9146781 10.1046/j.1365-2036.1997.142318000.x

[21] Svedlund J, Sjodin I & Dotevall G (1988) GSRS: a clinical rating scale for gastrointestinal symptoms in patients with irritable bowel syndrome and peptic ulcer disease. Dig Dis Sci 33, 129–134. 3123181 10.1007/BF01535722

[22] Lee W, Hamernyik P, Hutchinson M, (1982) Ascorbic acid in lymphocytes: cell preparation and liquid chromatography assay. Clin Chem 28, 2165–2169. 7127749

[23] Johanson JF, Wald A, Tougas G, (2004) Effect of tegaserod in chronic constipation: a randomized double-blind, controlled trial. Clin Gastroenterol Hepatol 2, 796–805. 15354280 10.1016/s1542-3565(04)00356-8

[24] Leung L, Riutta T & Kotecha J, (2011) Chronic constipation: an evidenced based review. J Am Board Fam Med 24, 436–451. 21737769 10.3122/jabfm.2011.04.100272

[25] Vandeputte D, Falony G, Vieira-Silva S, (2016) Stool consistency is strongly associated with gut microbiota richness and composition, enterotypes and bacterial growth rates. Gut 65, 57–62. 26069274 10.1136/gutjnl-2015-309618 PMC4717365

[26] Dimidi E, Christodoulides S, Fragkos C, (2014) The effect of probiotics on functional constipation in adults: a systematic review and meta-analysis of randomised controlled trials. Am J Clin Nutr 100, 1075–1084. 25099542 10.3945/ajcn.114.089151

[27] Stonehouse W, Gammon CS, Beck K, (2013) Kiwifruit: our daily prescription for health. Can J Physiol Pharmacol 91, 442–447. 23746068 10.1139/cjpp-2012-0303

[28] Zhao Y & Yu YB (2016) Intestinal microbiota and chronic constipation. SpringerPlus 5, 1130. 27478747 10.1186/s40064-016-2821-1 PMC4951383

[29] Wallace A, Eady S, Drummond L, (2017) A pilot randomised cross-over trial to examine the effect of kiwifruit on satiety and measures of gastric comfort in healthy adult males. Nutrients 9, 639. 10.3390/nu9070639 PMC5537759 28640214

[30] Rome Foundation (2016) Rome IV Functional Gastrointestinal Disorders. Raleigh, NC: Rome Foundation http://theromefoundation.org (accessed January 2016).

[31] Rajindrajith S, Devanarayana NM & Benninga MA (2017) Constipation and constipation predominant irritable bowel syndrome: a comparative study using Rome III criteria. J Paed Gastroenterol Nutr 64, 679–684. 10.1097/MPG.0000000000001332 27403609

[32] Rolls BJ, Bell EA, Castellanos VH, (1999) Energy density but not fat content of foods affected energy intake in lean and obese women. Am J Clin Nutr 69, 863–887. 10232624 10.1093/ajcn/69.5.863

[33] Rolls BJ, Ello-Martin JA & Tohill BC (2004) What can intervention studies tell us about the relationship between fruit and vegetable consumption and weight management? Nutr Rev 62, 1–17. 14995052 10.1111/j.1753-4887.2004.tb00001.x

[34] Ministry of Health (2015). Eating and Activity Guidelines for New Zealand Adults. Wellington: Ministry of Health.

[35] Shridhar G, Rajendra N, Murigendra H, (2015) Modern diet and its impact on human health. J Nutr Food Sci 5, 430–433.

[36] Thompson FE, Subar AF, Loria C, (2010) Need for technological innovation in dietary assessment. J Am Diet Assoc 110, 48–51. 20102826 10.1016/j.jada.2009.10.008 PMC2823476

[37] Dehghan M, Akhtar-Danesh N, McMillan CR, (2007) Is plasma vitamin C an appropriate biomarker of vitamin C intake? A systematic review and meta-analysis. Nutr J 6, 41. 17997863 10.1186/1475-2891-6-41 PMC2200644

[38] Carr AC, Pullar JM, Moran S, (2012) Bioavailability of vitamin C from kiwifruit in non-smoking males: determination of ‘healthy’ and ‘optimal’ intakes. J Nutri Sci 1, e14. 10.1017/jns.2012.15 PMC4153093 25191543

[39] Ranganathan P, Pramesh CS & Aggarwal R (2016) Common pitfalls in statistical analysis: intention-to-treat versus per protocol analysis. Perspect Clin Res 7, 144–146. 27453832 10.4103/2229-3485.184823 PMC4936074

[40] Liu KS & Mager D (2016) Women's involvement in clinical trials: historical perspective and future implications. Pharm Pract 14, 708. 10.18549/PharmPract.2016.01.708 PMC4800017 27011778

[41] Longstreth GF, Thompson WG, Chey WD, (2006) Functional bowel disorders. Gastroenterology 130, 1480–1491. 16678561 10.1053/j.gastro.2005.11.061

[42] Lee SY, Kim JH, Sung I, (2007) Irritable bowel syndrome is more common in women regardless of the menstrual phase: a Rome II based survey. J Korean Med Sci 22, 851–854. 17982234 10.3346/jkms.2007.22.5.851 PMC2693852

[43] Banks I (2001) No man's land: men, illness and the NHS. Br Med J 323, 1058–1060. 11691768 10.1136/bmj.323.7320.1058 PMC1121551

[44] Bazzano LA, Joshipura KJ, Li TY, (2008) Intake of fruit, vegetables and fruit juices and risk of diabetes in women. Diabetes Care 31, 1311–1317. 18390796 10.2337/dc08-0080 PMC2453647

